# Tolerogenic dendritic cells pulsed with islet antigen induce long-term reduction in T-cell autoreactivity in type 1 diabetes patients

**DOI:** 10.3389/fimmu.2022.1054968

**Published:** 2022-11-23

**Authors:** Tatjana Nikolic, Jessica S. Suwandi, Joris Wesselius, Sandra Laban, Antoinette M. Joosten, Petra Sonneveld, Dick Mul, Henk-Jan Aanstoot, John S. Kaddis, Jaap Jan Zwaginga, Bart O. Roep

**Affiliations:** ^1^ Department of Internal Medicine, Leiden University Medical Center, Leiden, Netherlands; ^2^ Diabeter Nederland, Diabetes Center, Rotterdam, Netherlands; ^3^ Department of Diabetes and Cancer Discovery Science, Arthur Riggs Diabetes and Metabolism Research Institute at the Beckman Research Institute, City of Hope, Duarte, CA, United States

**Keywords:** type 1 diabetes, islet autoimmunity, clinical trial, immune intervention therapy, immunotherapy, tolerance induction, antigen-specific therapy

## Abstract

**Introduction:**

Restoration of immune tolerance may halt progression of autoimmune diseases. Tolerogenic dendritic cells (tolDC) inhibit antigen-specific proinflammatory T-cells, generate antigen-specific regulatory T-cells and promote IL-10 production *in-vitro*, providing an appealing immunotherapy to intervene in autoimmune disease progression.

**Methods:**

A placebo-controlled, dose escalation phase 1 clinical trial in nine adult patients with long-standing type 1 diabetes (T1D) demonstrated the safety and feasibility of two (prime-boost) vaccinations with tolDC pulsed with a proinsulin peptide. Immunoregulatory effects were monitored by antigen-specific T-cell assays and flow and mass cytometry.

**Results:**

The tolDC vaccine induced a profound and durable decline in pre-existing autoimmune responses to the vaccine peptide up to 3 years after therapy and temporary decline in CD4 and CD8+ T-cell responses to other islet autoantigens. While major leukocyte subsets remained stable, ICOS^+^CCR4^+^TIGIT^+^ Tregs and CD103^+^ tissue-resident and CCR6^+^ effector memory CD4^+^ T-cells increased in response to the first tolDC injection, the latter declining thereafter below baseline levels.

**Discussion:**

Our data identify immune correlates of mechanistic efficacy of intradermally injected tolDC reducing proinsulin autoimmunity in T1D.

## Introduction

Induction or restoration of immune tolerance has been the ultimate aspiration in transplantation, allergy and autoimmune diseases (1). Many efforts over the past decades have been directed to control the underlying autoimmune process in type 1 diabetes (T1D) to inhibit beta-cell destruction. Preservation or restoration of endogenous insulin production is indeed essential for physiological blood glucose control reducing the risk of diabetic complications (2). Ideally, an immune intervention should selectively target beta-cell directed autoimmunity, leaving general immunity against, for instance, cancer and pathogens intact. Abundant evidence from preclinical studies has shown that direct oral, intra-nasal or parenteral administration of whole autoantigens, peptides or plasmids is safe (3–7), be it with minimal clinical benefits (8).

Dendritic cells (DC) add flexibility to immune modulation as they present antigens of choice to critically determine the quality and direction of T-cell activation combining antigen presentation with appropriate immunoregulating signals (9). When skewed towards a tolerogenic phenotype and presenting beta-cell autoantigens, DCs could initiate or activate a regulating immune response and dampen the existing immunity against these antigens. Indeed, our preclinical studies demonstrated mechanisms of action supporting these hypotheses. In humanized HLA-DR4-transgenic mice, proinsulin peptide-pulsed tolDC prevented and reversed induced autoimmunity to proinsulin that lasted upon subsequent challenges with the islet autoantigen (10). Human tolDC regulate adaptive immunity by antigen-specifically eliminating CD4^+^ and CD8^+^ T-cells and inducing antigen-specific Tregs (10–14). Tregs, in turn, change mature DC to become anti-inflammatory (‘infectious tolerance’) and suppress immune responses to other islet autoantigens present on the same DC (‘linked suppression’) (12). These processes proved critically dependent on expression of PD-L1, membrane-bound TNF, ICOS-L, B7-H3 and the appropriate HLA on tolDC to allow antigen-specificity. TolDC-induced Tregs resemble induced antigen-specific Tr-1, *in vivo* circulating islet-specific Tregs as well as (activated) thymic derived Tregs (tTregs) (15–17).

The choice of islet autoantigen to be used to as vaccine for the induction of tissue-specific Tregs is critical. We previously helped identifying a peptide derived from proinsulin and spanning the C-peptide region with the A-chain sequence of proinsulin (C19-A3) that is naturally processed and presented by APC by their high T1D-risk HLA-DR4 (18). Curiously, this self-peptide elicited proinflammatory responses in T1D patients carrying HLA-DR4, versus immune regulatory T-cell responses in HLA- and age-matched non-diabetic subjects, while T1D patients showing a combination of pro- and anti-inflammatory responses to this peptide developed T1D significantly later in life suggestive of an immune or disease modifying capacity (18). For this reason, and to avoid potential vaccine-induced allergic responses against whole insulin, we elected this peptide as our vaccine. Previous observations that injecting this naturally derived proinsulin peptide C19-A3 in humans is safe and that tolDC regulate antigen-specific immune responses *in vitro* prompted us to assess this peptide-cell combination for clinical intervention in T1D patients (6, 18, 19). After extensive preclinical characterization of tolDC production using a natural immunomodulator 1,25(OH)_2_vitaminD3 followed by dexamethasone and with ensuring robust stability and quality (20–23), these proinsulin-pulsed tolerance-inducing DCs were used in our recently completed phase 1 clinical trial (24). Nine patients with long standing T1D randomized into three groups each received one of the escalating doses (0.5x10^7^, 1.0 x10^7^ or 2.0 x10^7^ cells) of proinsulin-pulsed tolDC by intradermal injections twice as prime-boost one month apart (24). One patient in each dosing group was first injected intradermally with saline only (tolDC-vehicle) allowing monitoring of this placebo administration for three months prior to receiving peptide-pulsed tolDC injections in the respective dose. Safety and feasibility of this therapy was reported recently (24). Here, we report on mechanistic (immunological) efficacy of this approach to blunt islet autoimmunity. Building on our knowledge on their mechanisms of action of tolDC (21, 25), we examined the immunological effects of tolDC treatment *in vivo* using various analyses measuring adaptive immunity and high-dimensional phenotyping of leukocyte subsets in peripheral blood.

## Results

### TolDC treatment modulates T-cell autoimmunity to the vaccine peptide

**Figure 1:**
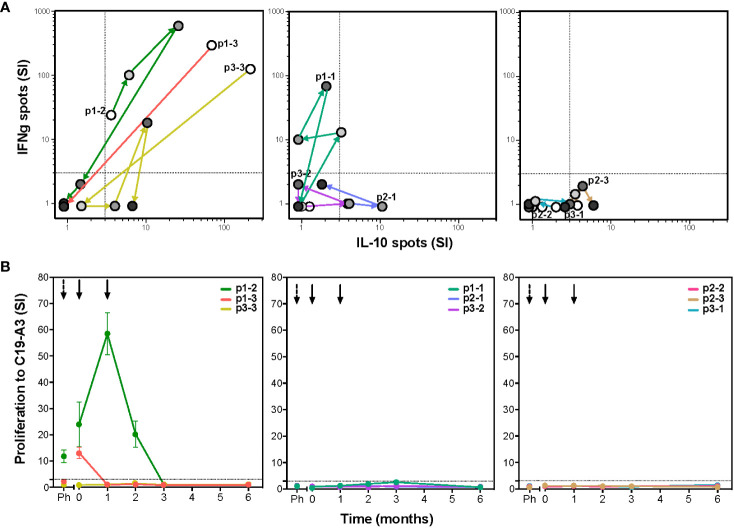
TolDC therapy modulates T-cell responses to the vaccine peptide (C19-A3) **(A)** The C19-A3-specific T-cells producing cytokines IFNg and IL-10 were quantified using the ELISPOT assay. Left graph shows changes in IFNg/IL-10 production in three patients (p1-2, p1-3 and p3-3) with detectable cytokine responses to C19-A3 prior to tolDC therapy. Middle graph shows responses in three patients (p1-1, p2-1 and p3-2) with a temporary cytokine production to C19-A3 and right graph shows responses in three patients (p2-2, p2-3 and p3-1) with very low responses to C19-A3 prior and after tolDC therapy. For each patient, white circles show the number of cytokine-producing cells prior to tolDC treatment, successive circles with increasing gray tones depict 1, 2, 3 and 6 months post-tolDC therapy values. Patients are coded according to the received tolDC dosage (p1-1 to p1-3 0.5x10^7^ cells; p2-1 to p2-3 1x10^7^ cells and p3-1 to p3-3 2x10^7^ cells). **(B)** Proliferative response to C19-A3 as determined by the lymphocyte stimulation test (LST). Proliferation was measured in triplicate as counts per minute (CPM) in wells with C19-A3 and stimulation index (SI) calculated as described in material and methods. Time of tolDC injections are indicated with black arrows.

To determine whether autoreactive responses were modulated after tolDC treatment, we evaluated *ex vivo* T-cell proliferation and cytokine production upon antigen stimulation. Seven out of nine patients showed changes in islet autoimmunity upon injection with C19-A3 pulsed tolDC (**Figure 1**). Patients were coded according to the received tolDC dosage per injection and order of administration (p1-1, p1-2 and p1-3 with 0.5x10^7^ cells; p2-1, p2-2 and p2-3 with 1x10^7^ cells; and p3-1, p3-2 and p3-3 with 2x10^7^ cells). T-cell autoimmunity to the vaccine peptide pre-existed before tolDC injection in three out of nine patients (p1-2, p1-3 and p3-3). Strikingly, all three responded to the tolDC therapy by reducing the proliferation (lymphocyte stimulation test (LST), **Figure 1B**) and cytokine production (ELISPOT, **Figure 1A**) to loss of responsiveness (cases p1-2 and p1-3) or IL-10 production alone (case p3-3) at the end of the 6-month study period. Of the six patients not reacting to the vaccine peptide before tolDC therapy, four showed a transient change in cytokine production (p1-1, p2-1, p2-3 and p3-2), with one patient (p2-3) attaining a moderate IL-10 producing phenotype at 6-month post-tolDC.

**Figure 2:**
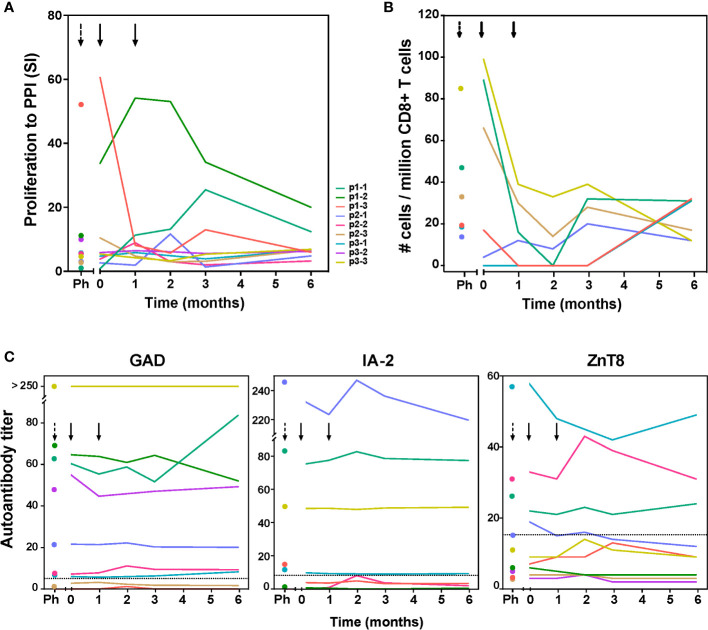
Immunity to preproinsulin (PPI) and autoantibody titers up to 6 months after tolDC therapy. **(A)** Proliferative responses of T-cells reactive with preproinsulin are depicted prior to leukapheresis (first time point), before tolDC (0) and at different time points after tolDC therapy for individual patients. **(B)** Quantification of PPI9-24-specific CD8**^+^** T-cells using Q-dot assay prior to leukapheresis (Ph), before tolDC (0) and at different time points after tolDC therapy in six HLA-A2**^+^** patients. In three patients with the highest PPI-specific T-cell counts prior to therapy, reduction after tolDC therapy was observed, while low numbers of PPI-specific CD8**^+^** T-cells pre-treatment, remained low. **(C)** Autoantibody titers (GAD65, IA-2 and ZnT8) after tolDC treatment, show no changes i.e. signs of induction/increase in beta–cell autoimmunity. Lines represent individual patient titers. Values in the gray area are below the negative cut-off for that specific autoantibody. Titers too low to show in the graphs: for p1-3 GAD ≤ 1.2; for p1-2 IA-2 ≤ 0.8, for p2-3 and p3-2 IA-2 = 0.

Next to the vaccine peptide, we measured autoreactive responses to preproinsulin protein (PPI) containing the vaccine peptide, and to other beta-cell autoantigens (GAD65 and IA-2; **Figure 2A**; **Figure S1**), and enumerated beta-cell autoreactive CD8^+^ T cells (**Figure 2B**; **Figure S2**) and auto-antibody titers to GAD, IA-2 and ZnT8 (**Figure 2C**). Before tolDC administration, inter-individual differences in proliferative response to autoantigens were noted in the frequencies of autoreactive PPI-specific CD8^+^ T-cells and autoantibody titers (**Figure 2**). Patients p1-2 p1-3 and p3-3 showed strong proliferative responses to PPI antigen (SI>10), while patients p1-1, p2-3 and p3-3 had PPI-peptide specific CD8^+^ T-cells before tolDC. Following tolDC administration, all patients presented with reduced values in both proliferative autoimmune responses and frequencies of autoreactive CD8^+^ T-cells, while autoantibody titers remained unaffected (**Figure 2**). The responses to IA-2 and GAD65 prior to therapy did not change significantly by 6 months after tolDC therapy (**Figure S1**) in 8 out of 9 patients, whereas patient p1-1 showed minimal proliferation to control antigen (tetanus toxoid; TetTox) and beta-cell autoantigens prior to tolDC injection, which could reflect a refractory period of lymphocytes shortly after leukapheresis. Indeed, this patient subsequently showed increasing proliferation to all tested antigens including TetTox control. Of note, CD8**^+^** autoreactivity and autoantibody titers in this patient were similar before leukapheresis and after tolDC injection.

### Long-term modulation of T-cell responsiveness to PPI and diabetes control after tolDC

**Figure 3:**
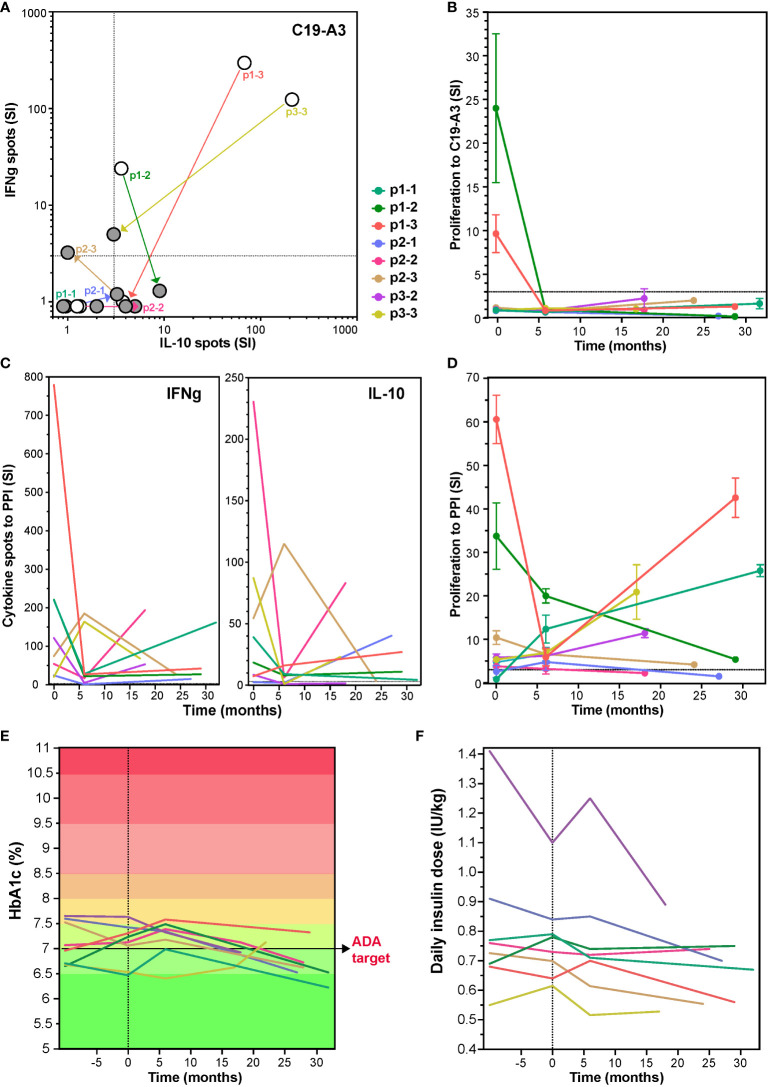
Change in T-cell responses to C19-A3 vaccine peptide, preproinsulin (PPI) and glycemia control at long term after tolDC therapy. Long-term effect of tolDC therapy on the cytokine response to C19-A3 **(A)** and PPI **(C)**. Patients reacting to C19-A3 prior to tolDC therapy, retain low response to C19-A3 at revisit and four out of eight patients show predominantly IL-10-producing response. TolDC therapy also reduced cytokine release to PPI with IFNg more strongly reduced than IL-10 in six out of eight patients at revisit (IFNg p=0.0012 and IL-10 p=0.34, paired T-test). White dots in **(A)** depict the cytokine spots before tolDC and filled dots at revisit. Patient specific values are connected. **(B)** and **(D)** Proliferation to C19-A3 peptide **(B)** and PPI **(D)** at revisit compared to the values before tolDC treatment. Lines in **(B)** to **(F)** represent individual patients coded in color as depicted. Patients p1-2, p1-3 and p2-1 reduced proliferation on the long term, other patients responses remain unchanged except for patient p1-1, showing an increased proliferation to PPI compared to the timepoint before tolDC therapy. **(E)** and **(F)** show change in HbA1c and insulin use in time.

To investigate long-term effects of the tolDC therapy, patients were invited to participate in a single re-call visit (17-32 months after receiving the tolDC injections), at which point the responses to the vaccination peptide, PPI antigen and diabetes control were again assessed in 8 patients available. Cytokine responses and proliferation to the vaccine peptide remained low or undetectable more than two years after the tolDC injection in all eight patients (**Figures 3A, B**). Moreover, IFNg production to PPI was long-term decreased in all patients as compared to the levels before tolDC injection (p=0.0016; **Figure 3C**). IL-10 showed more individual variation with a decrease in most patients except for p1-3 and p2-1. The strong proliferation to PPI (SI > 10) before tolDC in the case of p1-2, p1-3 and p3-3 (**Figure 3D**), decreased by 6 months after tolDC and persistently remained lower than before therapy. T-cell proliferation to other beta-cell antigens was also lower on the long term than at start in most patients (not shown).

Regarding glycemic control, all participants had long standing but well controlled T1D with HbA1c < 8% at start (range 6.4 – 7.6%), which was maintained after tolDC therapy and showed a clear decrease on the long term by an average of 0.34% (range 6.2 – 7.3%, p=0.0029, ANOVA), in spite of constant insulin needs (**Figures 3E, F**). Intriguingly, the HbA1c decline in some cases (p1-1, p2-1, p3-2, p3-3) resulted in personal ‘all-time low’ values long term, which was not always paired with detectable maintained endogenous insulin production (p2-2, p2-3, p3-2) (24).

### TolDC do not affect general immune profiles

**Figure 4:**
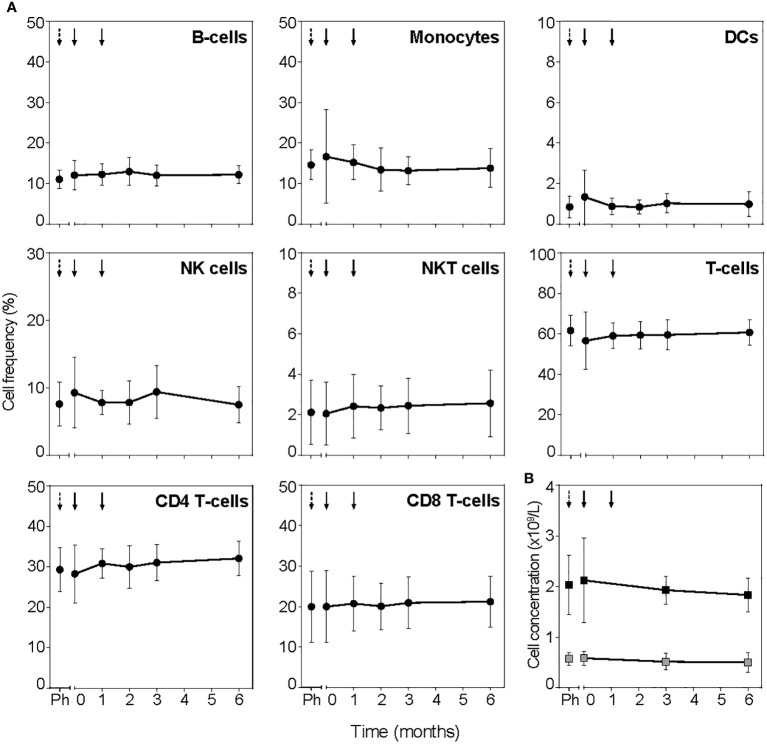
TolDC treatment does not change PBMC subsets. **(A)** Frequency of leukocyte subsets determined in full blood by flow cytometry. Timepoint before leukapheresis is indicated with black dashed arrow (Ph) and tolDC treatment with black arrows. **(B)** Lymphocytes (black squares) and monocytes (grey squares) determined in absolute cell counts. Symbols represent the mean value ± SD of all patients. TolDC treatments are indicated with black arrows, dashed arrow (Ph) indicates timepoint before leukapheresis.

To assess the general immune competence after tolDC treatment, total white blood cell counts and frequencies of leukocyte subsets measured in full blood by flow cytometry showed no systematic changes in total cell number or the frequency of leukocytes (B-cells, monocytes, DCs, NK cells, NKT cells, CD4**^+^** and CD8**^+^** T-cells; **Figure 4**; **Figure S3**). Further subdivision into subsets of B cells, monocytes, DC and NK cells did not show significant changes post tolDC treatment either (not shown). Antigen-specific immune competence unrelated to the tolDC-vaccine peptide or general islet autoimmunity (i.e. proliferation to TetTox and virus-specific CD8^+^ T-cell count) did not change for patients either in the placebo period or post tolDC treatment (**Figures S1** and **2**, respectively).

### Mass cytometry reveals changes in CD4^+^ and CD8^+^ T-cell clusters after tolDC treatment

**Figure 5:**
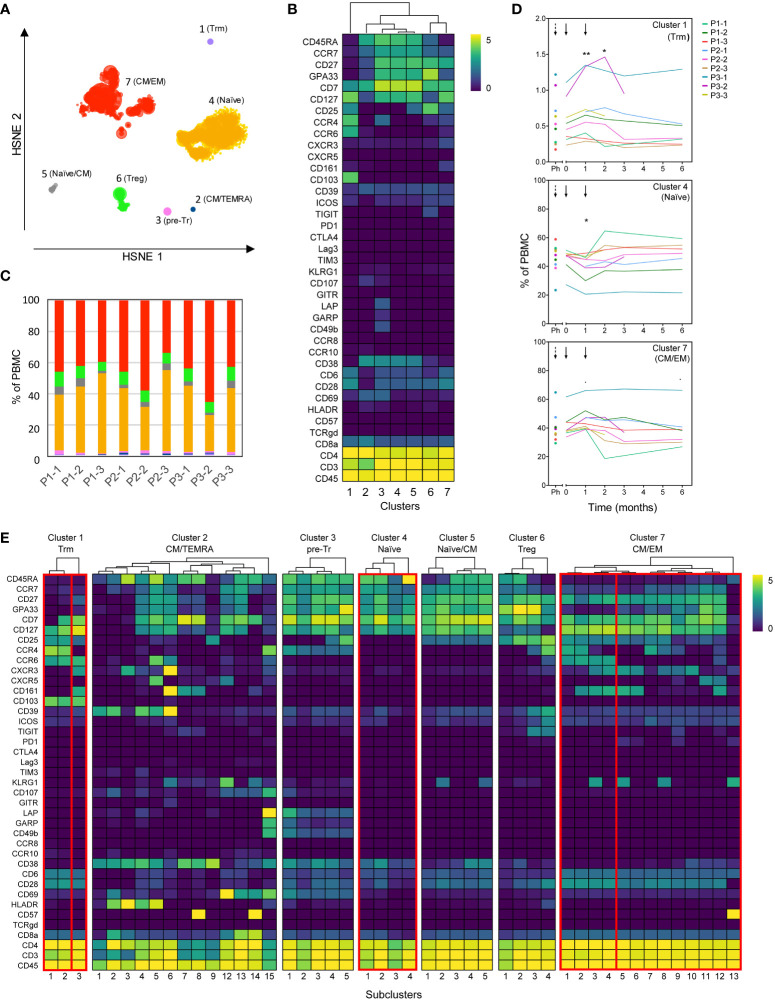
High dimensional analysis of the CD4^+^ T-cell compartment reveals changes after tolDC treatment. Analysis of CD4^+^ T-cells from tolDC-treated patients containing multiple timepoints before and after tolDC treatment (n=9). **(A)** HSNE analysis reveals seven major CD4**^+^** T-cell clusters at the overview level (level 1). **(B)** Clustered heatmap displays the phenotype of major clusters. **(C)** Distribution of major clusters within each patient (all timepoints combined). Colors correspond to the HSNE map in **(A)**. **(D)** Line plots display frequency of clusters for individual patients in time. The frequency in clusters 1, 4 and 7 significantly changed after tolDC treatment (cluster 1; p=0.012, cluster 4; p=0.007 and cluster 7; p=0.010). Black arrows show time of first and second tolDC injection, dashed arrows indicate the timepoint before leukapheresis. Each line represents one patient. **(E)** The heterogeneity within each major cluster is further explored in a new HSNE analysis (level 2). Generated subclusters are visualized in a heatmap. Boxes indicate significant major- and subclusters. Line plots with change in time of significant subclusters are displayed in **Figure S5**. Asterisks in **(D)** indicate significance levels for individual timepoints, *p<0.05 and **p<0.01.

Peep-diving into more subtle changes in minor subsets of immune cells, frozen PBMC sampled at all monitoring timepoints were stained simultaneously with a panel consisting of 39 lanthanide-conjugated antibodies (**Table S1**) and analyzed using Hierarchical Stochastic Neighbor Embedding (HSNE). Confirming the results obtained from fresh blood, no significant changes were observed in major leukocyte subsets or total CD4**^+^**, CD8**^+^** and TCRγδ**^+^** T-cell populations prior to or after tolDC treatment (**Figure S4**). Subsequent unsupervised HSNE analysis of the total CD4**^+^** T-cell population on the first level (level 1) allowed separation into seven major CD4-clusters based on the landmarks in the HSNE map (**Figure 5A**), with designations based on differential phenotypes (**Figure 5B**). While the cumulated frequencies of the clusters were similar between patients (**Figure 5C**), three out of seven clusters changed frequencies after tolDC administration compared to baseline (cluster 1; p=0.012, cluster 4; p=0.007 and cluster 7; p=0.010, ANOVA). Cluster 1 featuring a phenotype of tissue resident memory T cells (CD103^+^ Trm) showed an average increase of 0.13% (p=0.009) 1 month after the first tolDC injection and an increase of 0.10% 1 month after the second tolDC injection (p=0.047), compared to baseline value (0.56% of total CD4**^+^** T cells), after which the frequencies normalized to baseline (**Figure 5D**, top graph). Cluster 4, consisting of naïve CD4**^+^** T-cells (CD45RA^+^CCR7^+^) dropped from baseline (46.2% of total CD4**^+^**) by 4.5% at 1 month after the first tolDC injection (p=0.012). After the second tolDC injection, the frequencies of cluster 4 returned to baseline (**Figure 5D** middle graph). Cluster 7, comprised of effector CD4**^+^** T-cells with a mixed central and effector memory (CM/EM) phenotype (CD45RA^-^CCR7^dim^), showed an increase in most patients with an average of 3.9% at 1 month after the first tolDC injection, which declined after the second tolDC injection in many patients below the baseline level (40.5% of total CD4**^+^**) and remained low during the follow-up of 6 months after tolDC injection (**Figure 5D**).

**Figure 6:**
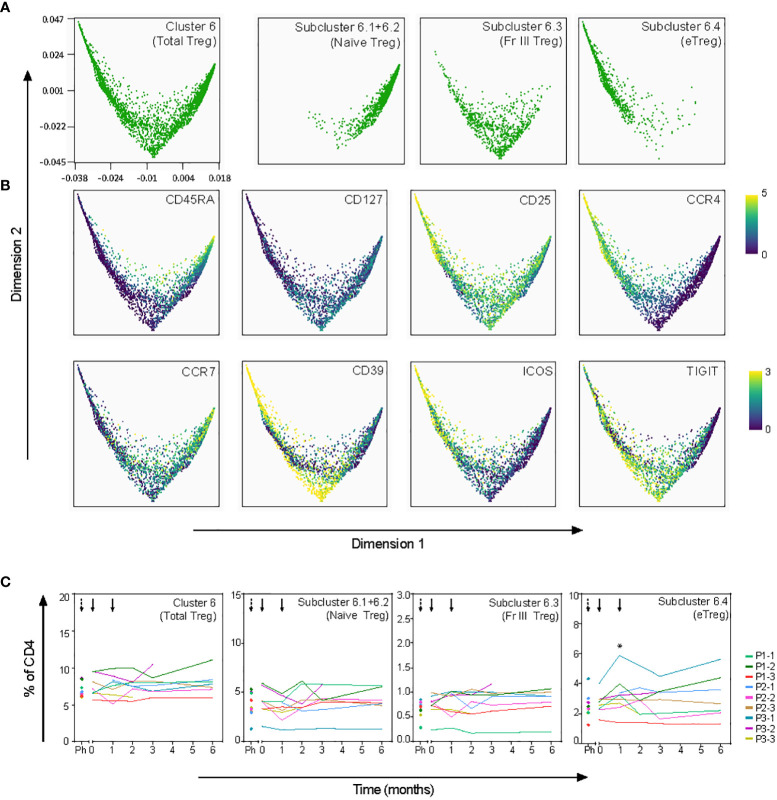
Effect of tolDC on regulatory T-cell subsets. Pseudotime analysis (diffusion map) shows differentiation trajectory within Treg cluster 6. **(A)** Treg subclusters as defined in Cytosplore (**Figure 5E**) are visualized in the diffusion map. Subclusters 6.1 and 6.2 are plotted together since both subclusters show a naïve phenotype. **(B)** Specified markers within the diffusion map. **(C)** Frequency in time of total Treg and Treg subclusters, shows significant increase of eTregs (subcluster 6.4) 1 month after tolDC treatment (*p=0.035).

To further explore the tolDC-induced changes, CD4**^+^** T-cell clusters 1, 4, 6 and 7 were analyzed in subclusters (level 2) (**Figure 5E**). Within the Trm cluster 1, one subcluster 1.3 demonstrated significant change (p=0.0003), though all Trm subclusters showed a peak in frequency after the first tolDC injection (**Figure S5A**). Within the CM/EM cluster 7, subclusters 7.1-7.4 shared expression of CCR6 and showed significant changes after tolDC injection (p=0.0003, p=0.003, p=0.0006 and p=0.001, respectively; **Figure 5E** and **Figure S5B**). Cluster 6 consisted of cells with a CD25^high^CD127^-^ phenotype and thus likely included both thymic derived Tregs and peripherally induced Tregs (26, 27). Although this Treg cluster showed no change in time overall, subdivision into four Treg subclusters (subcluster 6.1-4; **Figure 5E**) on basis of phenotype and pseudo-time analysis revealed changes corresponding with tolDC vaccination (**Figure 6**). These subclusters likely represent naïve Tregs expressing CD45RA (subcluster 6-1 and 6-2), an intermediate cluster previously described as Fraction III (28) and activated effector Tregs (eTregs; subcluster 6-4) expressing TIGIT, ICOS, CD39 and CCR4 (**Figures 6A, B** and **Figure S6**). Pseudo-time analysis showed a shift from naïve and Fraction III Tregs towards eTregs (subcluster 6-4) starting 1 month after the first tolDC injection with 2.7% eTregs at baseline increasing 0.5% on average by (p=0.035) at cost of the other Treg subclusters (**Figure 6C**).

A similar HSNE analysis of the CD8**^+^** T-cell compartment revealed eight major clusters (**Figure S7**). A significant change was detected in cluster 7 (p=0.042), representing CD8**^+^** T-cells with a memory phenotype (CD45RA^-^CCR7^dim^) expressing CD25^dim^ and the skin-tropic receptor CCR4 (**Figure S7D**). One month after the first tolDC injection, a 0.8% increase of CD8**^+^** was observed (p=0.0029), which normalized to baseline values (4.5% of total CD8**^+^**) after the second tolDC injection. Within cluster 7, subcluster 7.3 expressing KLRG1 (associates with T-cell senescence) increased significantly after tolDC injection (p=0.022) (29). Subclusters 7.1, 7.4 and 7.7 sharing low CD25 expression but lacking KLRG1 also showed a peak in frequency 1 month after the first injection (**Figure S7F**).

### Correlations between treatment, HbA1c, and immunologically relevant parameters in time

**Figure 7:**
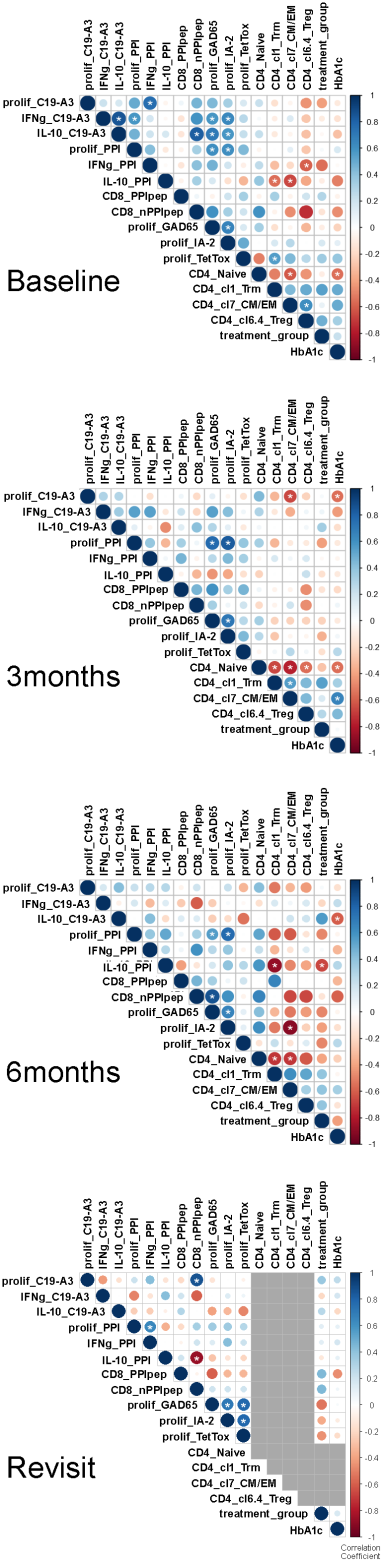
Correlation matrices at longitudinal time points. Multi-variable correlations between treatment dose, HbA1c, and immunologically relevant parameters. The size and color intensity denote the strength of the positive (blue) or negative (red) correlation; larger and darker, respectively, are stronger. Correlation scale ranges from 1 to -1 (perfect positive & negative, respectively). Grey used to represent missing data. Correlations of parameters previously identified by X were evaluated using Kendall’s rank correlation coefficient given the sample size and rank order nature of the method. In doing so, we applied a continuity correction since there were ties present in the data. All available patient data was used to determine correlation values. The R (build 3.6.3) package corrplot (version 0.84) was used to perform the analysis and visualize the results. All p-values are 2-sided. White asterisks indicate *p<0.05.

To study for relationships between clinical outcome with immune responses and T-cell subsets that associated significantly with tolerogenic vaccination, multi-variable correlations between treatment dose, HbA1c, phenotypic and immunological parameters were performed on all available data using Kendall’s rank correlation coefficient (**Figure 7**).

Before therapy, proliferative responses to PPI and vaccine peptide PPI_C19-A3_ correlated with cytokine responses to these autoantigens, as well as proliferative responses to other islet autoantigens (GAD65 and IA-2), but not with immune response to TetTox (**Figure 7**). Treg cluster 6.4 was inverse correlated with IFNg production in response to PPI as expected if this Treg subset suppresses autoimmunity. Likewise, the CD4 T effector clusters 1 (Trm) and 7 (CM/EM) inversely correlated with the production of cytokine IL-10 in response to PPI.

Three months after the first vaccination, correlations between cytokine responses to vaccine-specific and other autoantigens disappeared, only leaving correlations between proliferative response to PPI with IA-2 and GAD65. Instead, inverse correlation appeared between proliferation to the vaccine peptide and CD4 effector T-cell cluster 7, and to a lesser extent with HbA1c. The correlations further changed at 6 months when the correlation of effector T-cell subsets with proliferation to the PPI-vaccine peptide at 3 months transited into an inverse correlation with IL-10 production to PPI protein, supporting the induction of PPI-specific immune regulation following immunization with the PPI peptide. CD4 effector T-cell cluster 7 correlated inversely with autoimmunity to IA-2, while higher IL-10 response to the vaccine peptide associated with lower (i.e., improved) HbA1c values.

At time of the revisit (17-32 months after first vaccination and 11-26 months after completion of the clinical trial), the changes in correlations between immune responses to autoantigens compared to baseline persisted, with additional high IL-10 responses to PPI now correlating with reduced frequencies of CD8 T-cells against PPI.

## Discussion

With the safety and feasibility of tolDC therapy established in a phase 1 placebo-controlled dose-escalation study, we here describe immunological efficacy in our patients with long standing T1D (24). Notwithstanding the limited size of a safety trial, we obtained the first evidence of antigen-pulsed tolDC to modulate islet-cell autoimmunity *in vivo*, predominantly targeting CD4^+^ T-cell responses and sparing general immune reactivity. Prime-boost intradermal injections of tolDC selectively stimulated T-cells, including a subset of Tregs, and reduced the frequency of CCR6^+^ effector CD4^+^ T-cells, leaving all other peripheral blood cells unchanged. Furthermore, we found evidence of long-term antigen-specific immunological tolerance induced by tolDC in all patients with pre-existing vaccine-specific T-cell responses. Analyses of the autoimmunity more than two years after tolDC administration in a *post-hoc* patient revisit confirmed a long-lasting reduction in effector immune responses to the vaccine-peptide that in some cases extended to the whole PPI antigen. Patients also presented with long-term and stable (three patients) or improved glycemic control (five patients) despite similar or slightly reduced insulin need, compared to a 6-9-months monitoring period before the tolDC injection. While we have no definitive proof that these ‘all-time low’ HbA1c values are a direct consequence of tolDC immunotherapy, the superior glycemic control can be considered as important confirmation of the safety of our therapy.

In addition to the observed autoantigen-specific immune-modulation by tolDCs, high-dimensional phenotypic analysis of PBMC enabled discovery of specific T-cell subsets showing temporary changes that corresponded with TolDC vaccination. Increases in Treg subset *in vivo* resembled Tregs induced by tolDC *in vitro* (CD25, CCR4, ICOS and HLA-DR expression that associated with strong suppressive capacity) (17). TolDC therapy was also followed by a transient increase of Trm CD4**^+^** T-cells expressing CD103 (30). Trm cells can exit the tissue and re-enter the circulation (31). This transient increase might therefore reflect the response of skin-residing Trm to the intradermally injected tolDCs. Indeed, two patients receiving the highest tolDC dose (p3-1 and p3-2) showed the highest increase in Trm T-cells in blood. Most pronounced changes were observed for CCR6^+^ effector CD4**^+^** T-cells that initially increased but then declined at 6 months after tolDC therapy to frequencies below baseline values. TolDC induce antigen-specific apoptosis of effector CD4^+^ T-cells (12, 14). Within this declining effector T-cell pool, CCR6^+^ cells co-expressed CCR4 (subclusters 7.1 and 7.2) or CXCR3 (subclusters 7.3 and 7.4), indicating that levels of both Th17- and Th1-like Th17 cells may be reduced by tolDC therapy, both of which have been proposed to associate with beta-cell destruction and T1D (32–36). Likewise, monitoring of beta-cell specific autoimmunity by proliferation and ELISPOT analyses suggest that circulating effector T cells reactive to proinsulin peptide disappeared or became suppressed by IL-10 producing autoantigen-specific Tregs. This selective reduction in T-cell autoimmunity to islets persisted long after tolDC therapy in patients that showed pre-existing response (p1-2, p1-3, p3-3) and in an additional four patients, in which tolDC induced temporary immunity to the vaccine peptide (p1-1, p2-1, p2-3, p3-2). Multivariate analyses indicate persistent changes in correlations between islet autoimmunity and specific T-cell subsets supporting the induction of durable and specific immune regulation by tolDC vaccination. We speculate that short-lived proinflammatory autoimmunity to vaccine peptide only observed at 3 months may reflect immune activation that is required for the induction of immune regulation. We therefore propose that tolDC induce a form of immune tolerance to the vaccine peptide *in vivo*, warranting phase-2 studies in C-peptide positive patients to assess efficacy to preserve this functional beta-cell reserve.

TolDC therapy did not induce general changes within the CD8**^+^** T-cell compartment with only a transient and moderate increase of one cluster (cluster 7; CD25^dim^ memory CD8**^+^** T-cells). This heterogeneous CD8 T-cell subset contains cells with skin-homing and senescent phenotypes, as well as early effector CD8**^+^** T-cells (37). This would be consistent with our expectation that pulsing tolDC with a peptide eluted from HLA-DR4 primarily targets the CD4**^+^** T-cell compartment (18). Nonetheless, a specific and long-standing reduction of autoreactive CD8**^+^** T-cells was observed while virus-specific CD8**^+^** T-cells remained constant. In addition, the *ex vivo* proliferative response to other beta-cell antigens declined in time. While this could be a reflection of a general reduction in chronic inflammation and cytokine levels, we favor the possibility of linked suppression of other islet-specific responses, which is supported by increased levels of induced Tregs *in vivo*. These observations are not definitive but do warrant future testing of the potential of tolDC to induce infectious tolerance *in vivo*, as tolDC were demonstrated to induce *in vitro* (12).

The inherently limited phase 1 trial in long standing T1D precludes assessing clinical efficacy of the tolDC to preserve beta-cell function. Yet, our findings extend the safety of this candidate immune intervention strategy by showing stable blood leukocyte subsets and sparing of immune responses to virus and previous vaccines. Instead, selective modulation of relevant T cell subsets and a long-lasting annihilation of preexistent vaccine-specific responses to islet autoantigen were observed after the tolDC treatment, in combination with exceptional glycemic control long after therapy. TolDC therapy therefore seems able to adjust the immune system towards autoantigen-specific tolerance *in vivo*, justifying further clinical testing whether proinsulin-pulsed tolDC induce long-lasting specific immune modulation in T1D patients with remaining beta-cell function, to assess whether these functional beta-cells can be protected from autoimmune destruction.

## Materials and methods

### Study design

The clinical study protocol was approved by the Dutch Central Committee on Research Involving Human Subjects and the Medical Ethical Committee of Leiden University Medical Center (LUMC; Leiden, The Netherlands), EudraCT number 2013-005476-18 (accessible through the national trial registry). Patients were selected for screening by their own physician at Diabeter clinic (The Netherlands). Nine HLA-DR4 positive patients eligible for the study and willing to participate were allocated at random to a dose treatment group: 0.5x10^7^ (p1-1, p1-2 and p1-3), 1x10^7^ (p2-1, p2-2 and p2-3) or 2x10^7^ (p3-1, p3-2 and p3-3) tolDCs per injection round (24). A randomly selected patient in each dose-cohort first received 5, 10 or 20 intradermal injections of saline (the tolDC vehicle), respectively. In this ‘placebo’ period, participants were monitored for three months (12 weeks), after which they received tolDC injections and were monitored as a third participant in their respective dose-group.

### *In vitro* generation and administration of clinical grade tolDCs

Detailed description of clinical tolDC quality assessment has been reported (24). TolDCs were administered twice (prime/boost) by intradermal injection in the upper-left abdominal quadrant, using a MicronJet600 microneedle (NanoPass Technologies Ltd, Nes Ziona, Israel), at the Clinical Cellular Research Unit of the Hemapheresis Unit (LUMC) (24).

### Immunomonitoring

Antigen-specific immunomonitoring was performed using techniques previously validated for clinical trials: fresh PBMCs were cultured with GMP-grade C19-A3 peptide, or PPI protein to detect antigen-specific IFN-g and IL-10 producing cells using ELISPOT assay (18); antigen-specific proliferation was assessed in the lymphocyte stimulation test (LST) (38, 39). In short, fresh PBMC were incubated with GMP-grade C19-A3 peptide, recombinant human proteins PPI, GAD65 and IA-2 (all at final conc. 10 µg/mL), synthesized at the Protein Facility (LUMC). For control, cells were incubated with medium alone (med), tetanus toxoid (1%, 7.23 Lf/mL; Statens Serum Institut) or recombinant IL-2 (Proleukin^®^, 35 U/mL; Novartis, Bazel, Switzerland). Stimulation index (SI) is calculated as cpm in the presence of antigen/stimulus divided by cpm with medium alone. To quantify PPI-specific CD8^+^ autoreactive T-cells, the Q-dot assay was performed using frozen PBMC as described and validated previously (7, 40). PBMC subsets were measured using whole blood flow cytometry. Cryopreserved PBMC were used for the CyTOF analysis. Samples were stained with a panel consisting of 39 metal-conjugated antibodies (**Table S1**) as described previously (41). To minimize inter-assay variability, samples of different timepoints from the same patient were stained and acquired on the same day and a reference sample was included as quality control. The reference sample consisted of 1,5% PHA stimulated PBMC from a healthy donor. To check the consistency between staining and measurement days, reference samples were analyzed in a tSNE using Cytosplore and Jensen-Shannon plots were generated in Matlab (version R2016a) (**Figure S8C**).

### Statistical analysis

Proliferation in LST was tested using 2-way ANOVA and Dunnet’s multiple comparison test. Changes in ELISPOT were tested using Wilcoxons matched-pairs signed rank test. Flow cytometric analyses were performed using FlowJo software (Ashland, OR, USA). Principal component analysis was performed using R (package 3.5.2, ‘ggplot2’). Graphs and statistical calculations were performed using GraphPad software (San Diego, CA, USA). Values at p<0.05 after multiple testing correction were deemed significant.

Data obtained by CyTOF were analyzed as follows: Beads were excluded from the dataset and live, CD45^+^ single cells were selected with DNA stains and Gaussian parameters (width and residual) (**Figure S8A**) in FlowJo. Expression values were arcsine transformed and Hierarchical Stochastic Neighbor Embedding (HSNE) implemented in Cytosplore (version 2.2.1) (42) was used for dimensionality reduction analysis. The dataset was explored in different levels of hierarchies as described in the results section. Subsequent clusters were generated using the Gaussian-mean-shift method (**Figure S8B**). FCS files from generated clusters were exported and loaded in R (version 3.6.2) using the “Cytofast” package (43) for further downstream analysis. A mixed linear model was used to evaluate changes in cell frequency of specific clusters after tolDC treatment. Cell frequencies were compared with the baseline frequency at timepoint 0, before the first tolDC injection. The cell frequency of the cluster is selected as dependent variable, timepoints after tolDC injections are the fixed effects and the random effects are the different patients. An ANOVA test was used to assess whether overall changes are observed in a cluster after tolDC treatment and p-values below 0.05 were considered statistically significant. Estimates of fixed effects from the mixed linear model are reported in the results as change in percentage of CD4^+^ or CD8^+^ T-cells. Statistical tests were performed using R package “lmerTest”. Diffusion maps were generated in the OMIQ Data Science Platform using the Wanderlust function.

## Data availability statement

The original contributions presented in the study are included in the article/**Supplementary Material**. Further inquiries can be directed to the corresponding author.

## Ethics statement

The studies involving human participants were reviewed and approved by the Dutch Central Committee on Research Involving Human Subjects (CCMO, dossier number NL48984.000.14). The patients/participants provided their written informed consent to participate in this study.

## Author contributions

Conceptualization, TN, PS, JZ, and BR. Methodology, TN, PS, JS, and SL. Investigation, TN, JS, JW, SL, and AJ. Validation, TN, JW, and SL. Formal analysis, TN, JS, SL, and JW. Resources, DM and H-JA. Visualization, TN, JS, and JW. Statistical analyses, JK. Writing, TN, JS, JZ, and BR. Supervision, BR and JZ. All authors contributed to the article and approved the submitted version.

## Funding

This work was supported by the European Union (FP7-NAIMIT, Grant 241447), the Dutch Diabetes Research Foundation and Stichting DON (grant number 2020.10.011) and the Wanek Family Project for Type 1 Diabetes.

## Acknowledgments

We thank Koen Stam and Guillaume Beyrend for statistical analysis of the mass cytometry data, and Drs. Derk Amsen and Rianne Opstelten for providing the GPA33 antibody for the CyTOF panel.

## Conflict of interest

The authors declare that the research was conducted in the absence of any commercial or financial relationships that could be construed as a potential conflict of interest.

## Publisher’s note

All claims expressed in this article are solely those of the authors and do not necessarily represent those of their affiliated organizations, or those of the publisher, the editors and the reviewers. Any product that may be evaluated in this article, or claim that may be made by its manufacturer, is not guaranteed or endorsed by the publisher.
