# Supplementary material for: Tolerogenic dendritic cells pulsed with islet antigen induce long-term reduction in T-cell autoreactivity in type 1 diabetes patients
Source: Front Immunol. 2022 Nov 23;13:1054968. doi: 10.3389/fimmu.2022.1054968 (PMC9728525; doi:10.3389/fimmu.2022.1054968)
Supplement: Supplementary file 1 [file DataSheet_1.pdf]

Supplementary Materials

Table S1. Staining panel for mass cytometry.

Marker	Metal	Clone	Dilution
CD3*	161Dy	UCHT1	1:600
CD4	145Nd	RPA-T4	1:100
CD6*	198Pt	BL-CD6	1:100
CD7	166Er	M-T701	1:800
CD8a	146Nd	RPA-T8	1:800
CD25	169Tm	2A3	1:100
CD27	167Er	O323	1:200
CD28*	171Yb	CD28.2	1:150
CD38	172Yb	HIT2	1:200
CD39*	162Dy	A1	1:100
CD45	89Y	HI30	1:150
CD45RA	Qdot655	MEM-56	1:300
CD49b*	149Sm	P1e6c5	1:50
CD69	144Nd	FN50	1:300
CCR10	138Nd	6588-5	1:40
CD103*	155Gd	Ber-ACT8	1:50
CD107 (LAMP)*	143Nd	H4A3	1:50
CD127	165Ho	AO19D5	1:300
CD152 (CTLA4)*	170Er	14D3	1:40
CD161	164Dy	HP-3G10	1:150
CD138 (CXCR3)	163Yb	G025H7	1:50
CD185 (CXCR5)	173Yb	J252D4	1:100
CD194 (CCR4)*	156Gd	L291H4	1:50
CD196 (CCR6)	141Pr	G034E3	1:100
CD197 (CCR7)	142Nd	G043H7	1:100
CD223 (Lag-3)	150Nd	11C3C65	1:40
CD278 (ICOS)	151Eu	C398	1:50
CD279 (PD-1)	175Lu	EH 12.2H7	1:50
CD357 (GITR)*	159Tb	621	1:40
CD366 (TIM3)	154Sm	F38-2E2	1:50
TIGIT	153Eu	MBSA43	1:50
Putrid (GPA33)*	158Gd		1:50
HLA-DR*	168Er	L243	1:800
LAP (TGF-b)*	174Yb	TW4-2F8	1:40
TCRgd	152Sm	11F2	1:50
KLRG-1*	160Gd	REA261	1:50
GARP*	176Yb	7B11	1:50

*self-conjugated

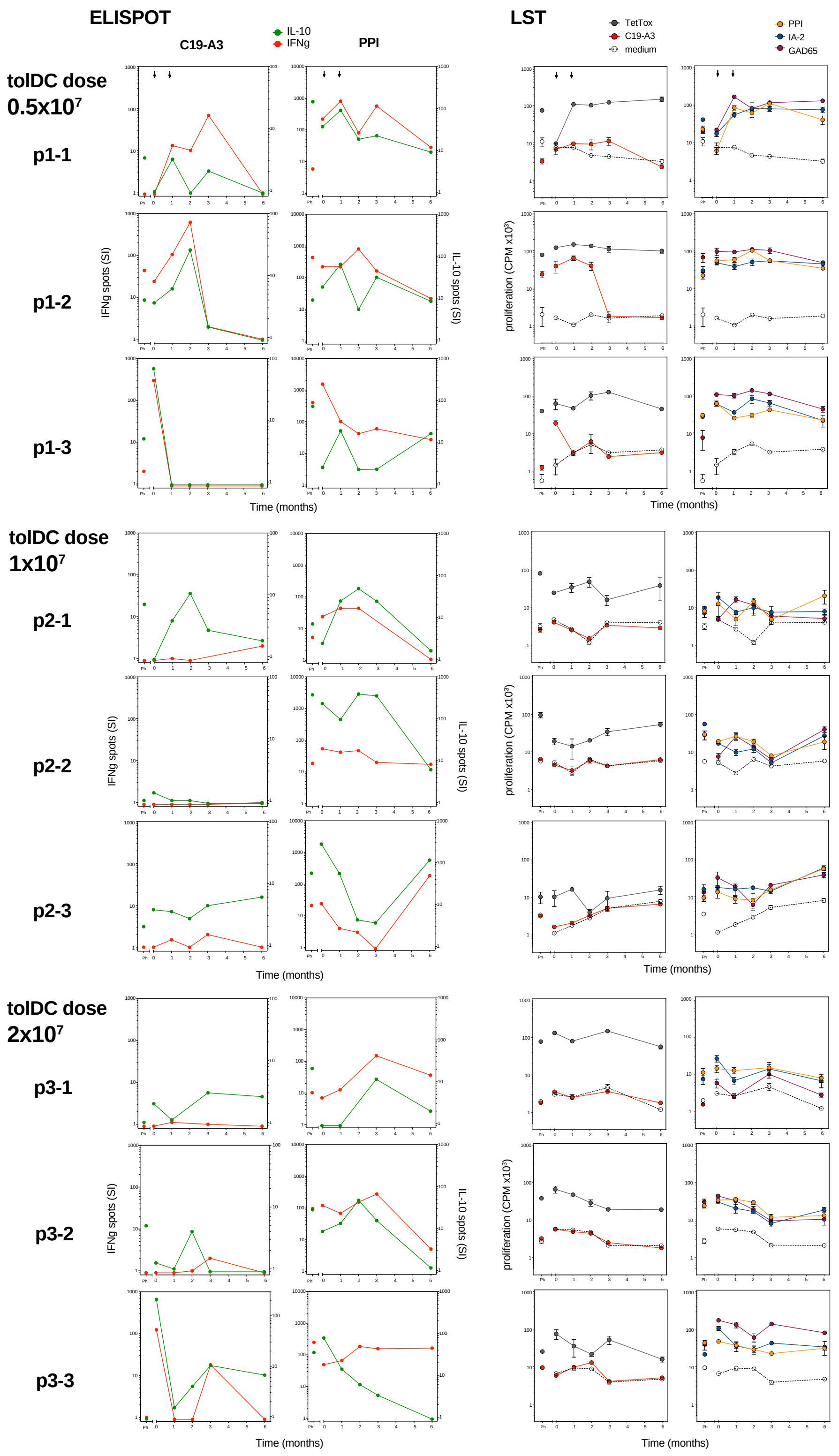

Fig. S1. Individual patient data for all monitoring timepoints (ELISPOT and LST): prior to leukapheresis (Ph), at tolDC injections (1st = 0; 2nd = 1 month) and at 2, 3 and 6 months after start of tolDC treatment. Arrows in the top row of graphs denote timepoints of tolDC injections. Patient graphs are grouped in panels based on the received tolDC dose (top panel = $5x10^6$; middle panel $10x10^6$; bottom panel $20x10^6$ per tolDC treatment). The left two columns depict cytokine producing cells, expressed in number of spots (green = IL-10 and red = IFNg), specific to the vaccine peptide (C19-A3) or whole proinsulin protein (PPI) as determined by the ELISPOT assay. Right two columns depict T cell proliferation (CPM) as measured by the lymphocyte stimulation test (LST). Red symbols/lines represent proliferation to C19-A3, grey = TetTox, yellow = PPI, blue = IA-2, dark red = GAD65. White symbols with dashed line represent background proliferation without antigen (medium control).

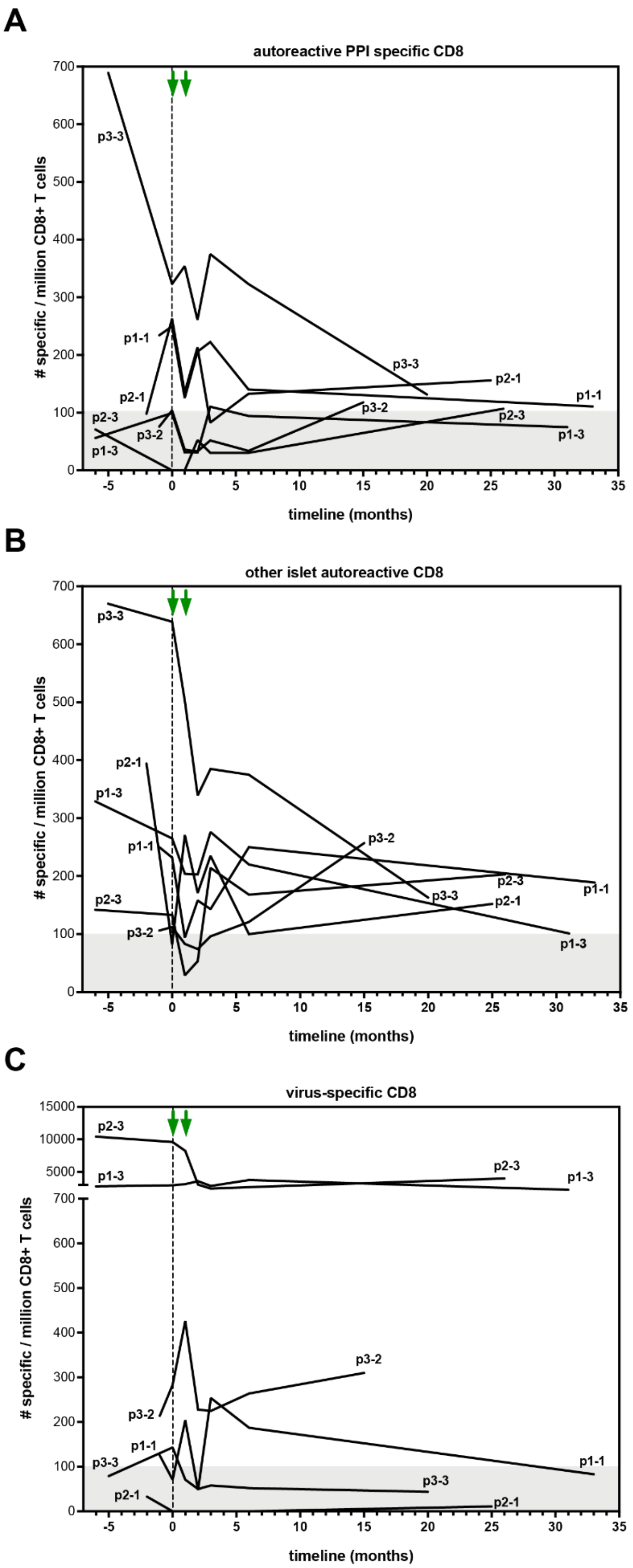

Fig. S2. Q-dot assay for quantification of antigen-specific CD8$^+$ T-cells. (A) Quantification of PPI-specific T-cells ($PPI_{15\text{-}24}$, $InsB_{10\text{-}18}$, INS-$DRIP_{1\text{-}9}$) using Q-dot assay prior to leukapheresis, before tolDC (0) and at different time points after tolDC therapy in six HLA-A2$^+$ patients. In three patients with the highest PPI-specific T-cell counts prior to therapy, reduction after tolDC therapy was observed, while low numbers of PPI-specific CD8$^+$ T-cells pre-treatment, remained low. **(B)** CD8 T-cells reactive to peptides from GAD65, IA-2, IGRP, IAPP and ZnT8 and **(C)** virus-specific CD8 T-cells, reacting to CMV, EBV or Measles virus epitopes.

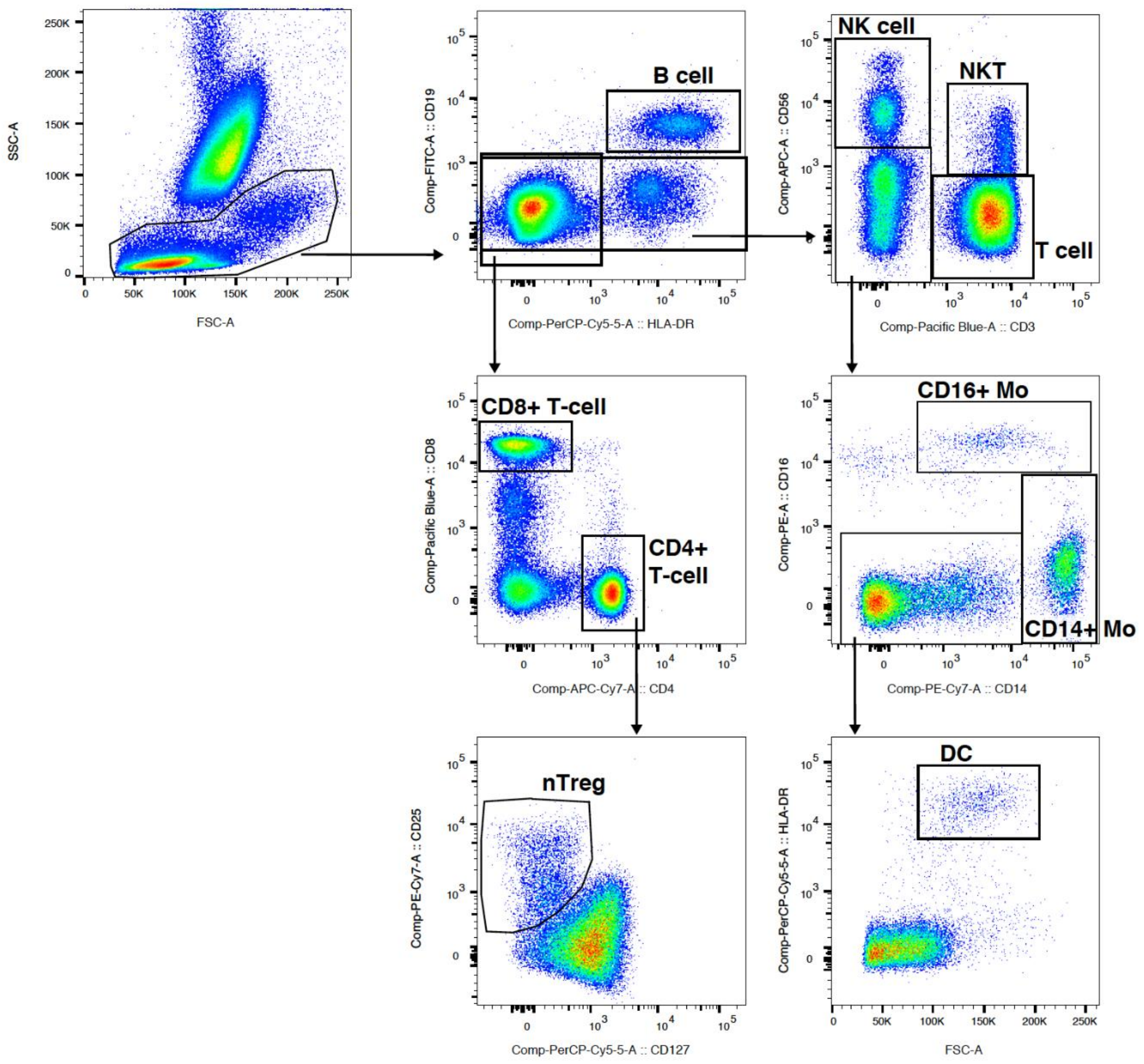

Fig. S3. Gating strategy of flow cytometry data. Major lineages from the innate and adaptive immune system from fresh blood samples are gated for comparison of quantities in time after tolDC treatment (Figure 4).

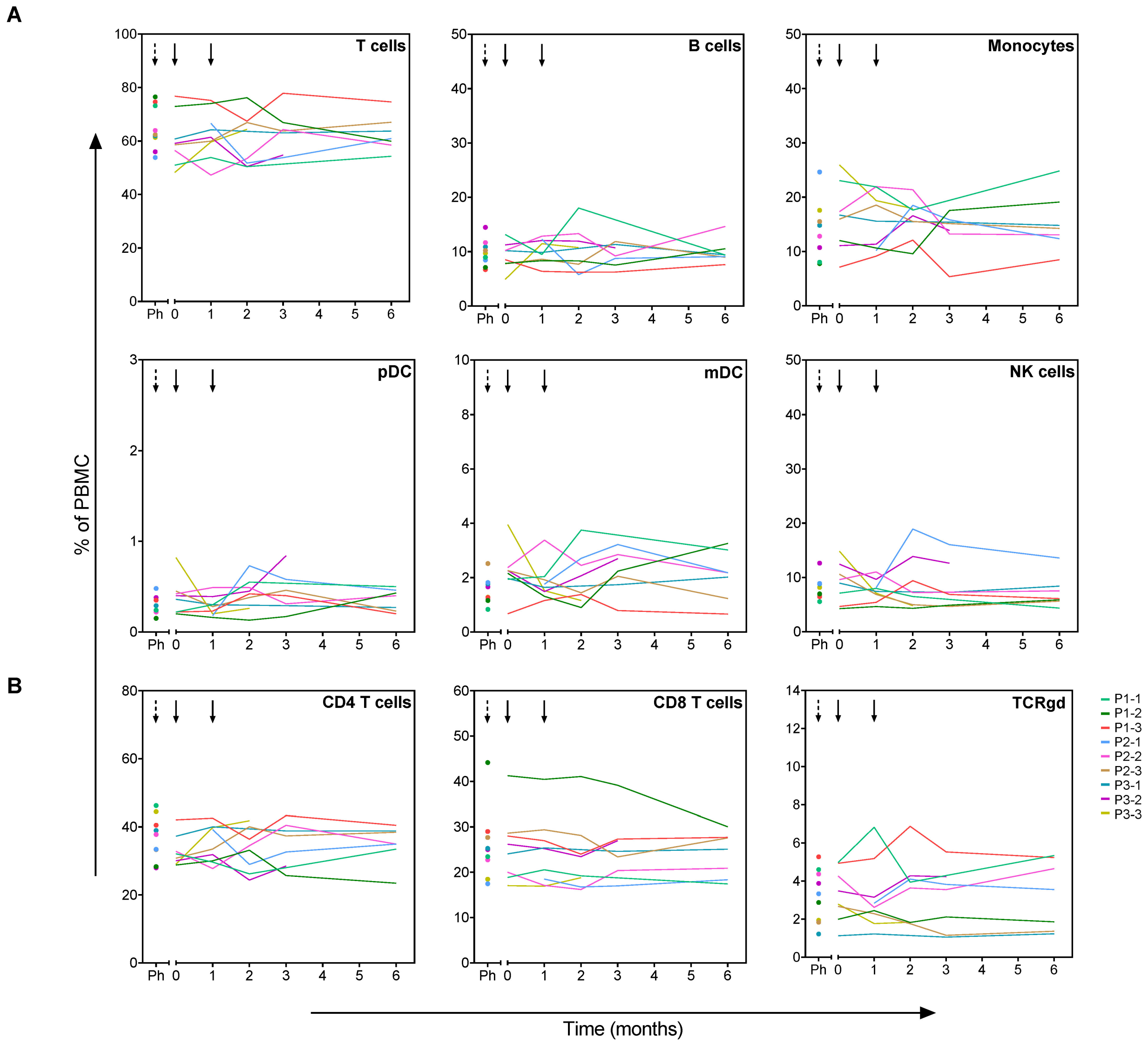

Fig. S4. Cell frequencies of leukocyte subsets in frozen PBMC. Frequencies determined by flow cytometry **(A)** and mass cytometry **(B)**. Timepoint before leukapheresis is indicated with black dashed arrow (Ph) and tolDC treatment with black arrows. No significant changes in major immune populations are observed after tolDC injections.

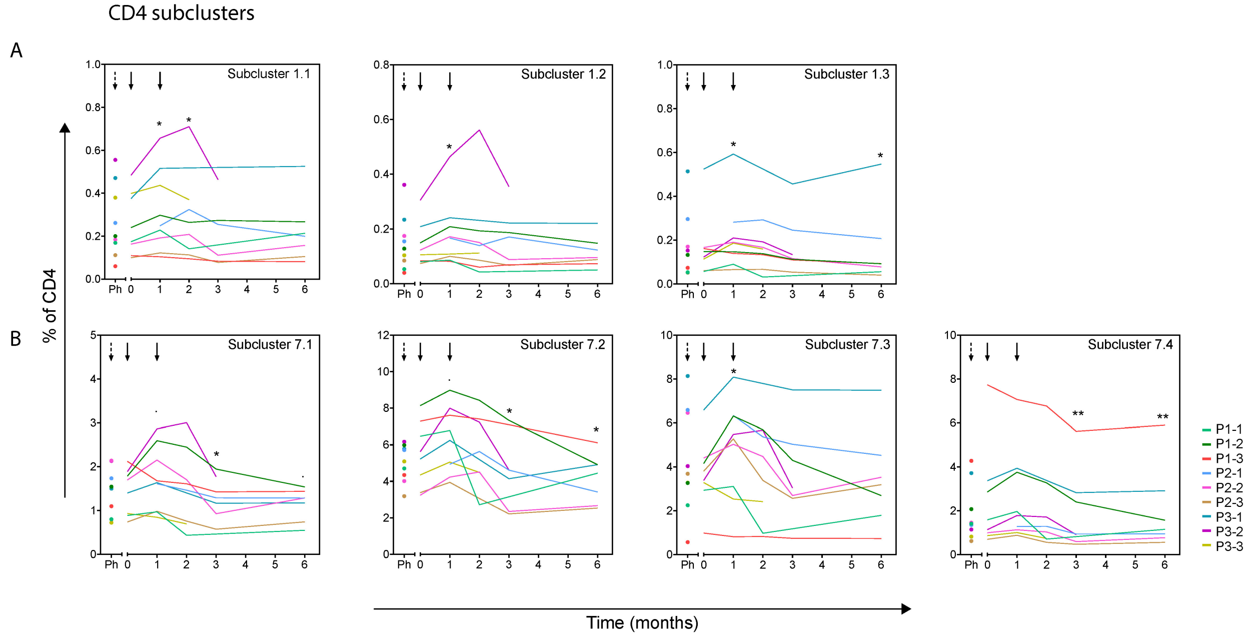

Fig. S5. Line plots of second level CD4+ T-cell subclusters that significantly change after tolDC injection. **(A)** Significant subclusters derived from Trm (CD103+) cluster 1 (1.1-1.3) and **(B)** memory cluster 7 with CCR6 expression (7.1-7.4), showing an increase 1 month after the 1st tolDC injection. Subcluster 7.1, 7.2 and 7.4 showed significant decrease after the 2nd tolDC injection compared to baseline frequencies.

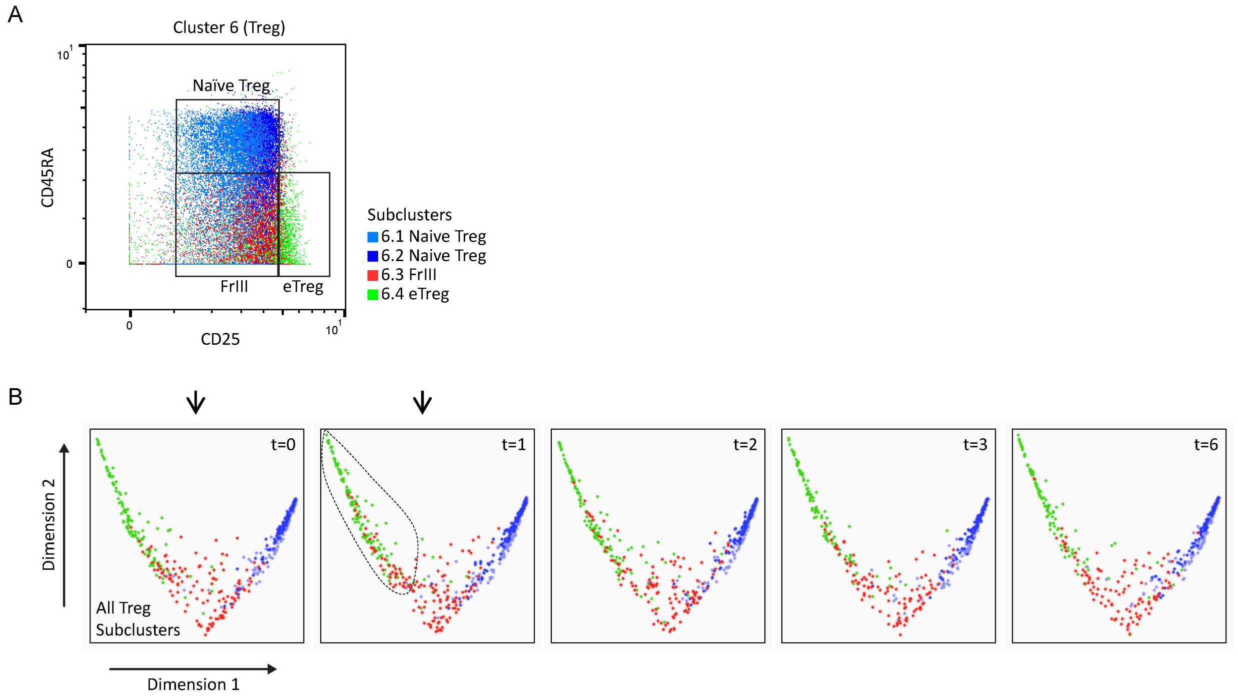

Fig. S6. Second level subclusters of Tregs (A) Treg subclusters as determined by Cytosplore visualized in conventional gating view for FACS analysis. **(B)** Diffusion map of Treg cluster 6. Cells from each timepoint are visualized in a different plot. Arrows indicate time of tolDC injection and time is indicated in months.

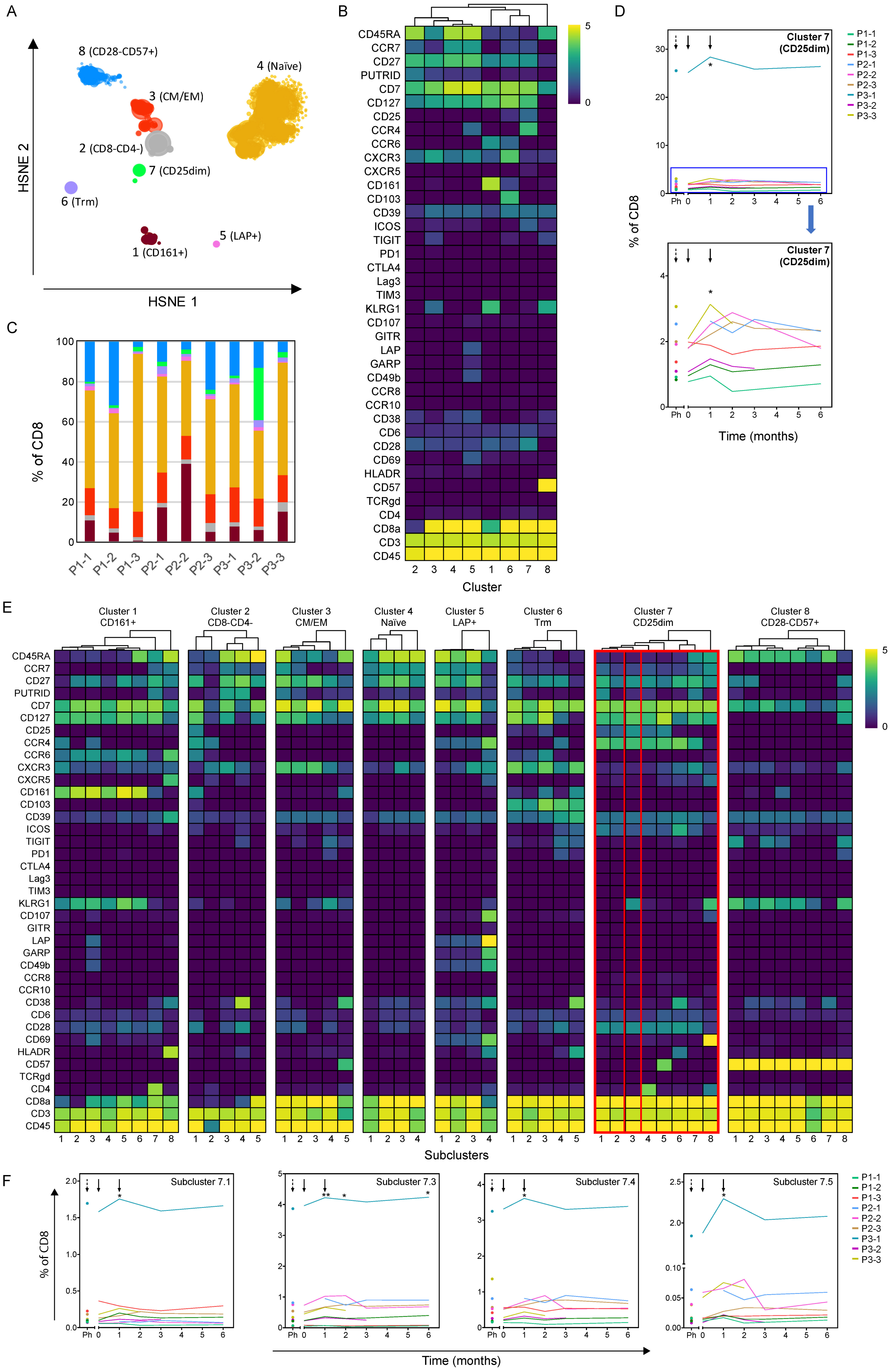
A
8 (CD28-CD57+)
3 (CM/EM)
4 (Naïve)
2 (CD8-CD4-)
7 (CD25dim)
6 (Trm)
5 (LAP+)
1 (CD161+)
HSNE 2
HSNE 1
B
CD45RA
CCR7
CD27
PUTRID
CD7
CD127
CD25
CCR4
CCR6
CXCR3
CXCR5
CD161
CD103
CD39
ICOS
TIGIT
PD1
CTLA4
Lag3
TIM3
KLRG1
CD107
GITR
LAP
GARP
CD49b
CCR8
CCR10
CD38
CD6
CD28
CD69
HLADR
CD57
TCRgd
CD4
CD8a
CD3
CD45
Cluster
C
% of CD8
P1-1
P1-2
P1-3
P2-1
P2-2
P2-3
P3-1
P3-2
P3-3
D
Cluster 7 (CD25dim)
% of CD8
Time (months)
E
Cluster 1 CD161+
Cluster 2 CD8-CD4-
Cluster 3 CM/EM
Cluster 4 Naïve
Cluster 5 LAP+
Cluster 6 Trm
Cluster 7 CD25dim
Cluster 8 CD28-CD57+
Subclusters
F
Subcluster 7.1
Subcluster 7.3
Subcluster 7.4
Subcluster 7.5
% of CD8
Time (months)

Fig. S7. CyTOF analysis of the CD8$^+$ T-cell compartment. Analysis of CD8$^+$ T-cells from different timepoints before and after tolDC treatment (n=9). **(A)** HSNE map of the CD8$^+$ T-cell compartment at the overview level (level 1) shows eight major clusters. **(B)** Heatmap visualizing the phenotype of the major CD8$^+$ T-cell clusters. **(C)** Distribution of major clusters within each patient (all timepoints combined). Colors correspond to clusters represented in the HSNE map in A. **(D)** Line plot of significant cluster 7, showing change in frequency after tolDC injection (p=0.0029). Each line represents one patient and arrows indicate time of tolDC injection. The bottom panel displays a close up of the boxed area in the left panel. **(E)** Heatmap of subclusters of CD8$^+$ T-cells. Boxes indicate significant clusters. **(F)** CD8$^+$ T-cell subclusters that significantly change after tolDC injection. Arrows indicate time of tolDC injection and each line represents one patient.

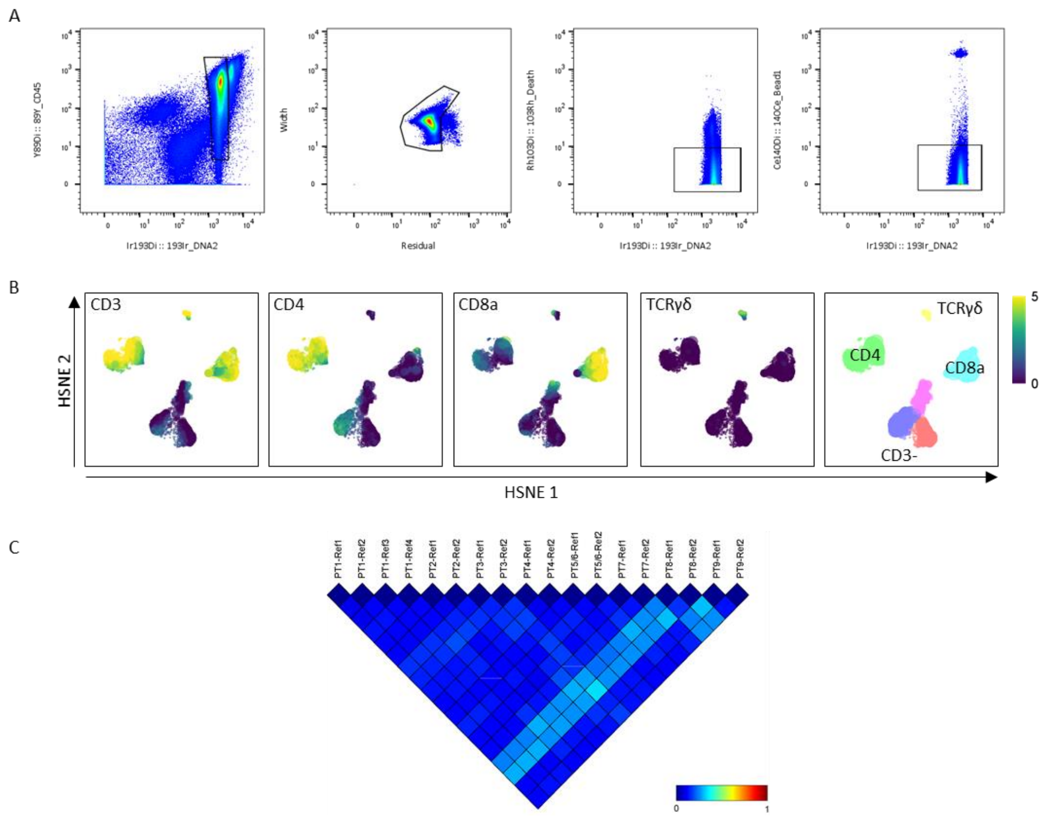

Fig. S8. Gating and quality control of CyTOF dataset. A) Gating strategy of CyTOF data to select for CD45⁺ cells. Doublets, dead cells and beads were excluded. B) T-cell populations were identified within CD45⁺ cells using HSNE analysis. C) Jensen-Shannon (JS) plot of reference samples visualizes the consistency of the staining between experiment days. Similarity between tSNE maps of reference samples are measured by the Jensen-Shannon divergence, values are shown between 0 (min) to 1 (max). Per staining, one reference sample was stained alongside the patient samples and subsequently split into different batches to measure at the start and end of each measurement day (1-2).
